# Interventions supporting meaningful connections for people with serious mental illness: a concept-framed systematic narrative review

**DOI:** 10.1007/s00127-025-02812-8

**Published:** 2025-01-18

**Authors:** Emi Patmisari, Yunong Huang, Mark Orr, Sumathi Govindasamy, Emily Hielscher, Helen McLaren

**Affiliations:** 1 College of Education, Psychology and Social Work, Flinders University, Adelaide, Australia; 2 Flourish Australia, Sydney Olympic Park, NSW Australia; 3 QIMR Berghofer Medical Research Institute, Herston, QLD Australia; 4 School of Public Health, Faculty of Medicine, The University of Queensland, Brisbane, QLD Australia; 5 School of Allied Health, Australian Catholic University, Melbourne, Australia

**Keywords:** CIVIC framework, Serious mental illness, Severe mental illness, SMI, Social connection, Social relationship

## Abstract

**Purpose:**

Meaningful connections, encompassing relationships providing emotional support, understanding, acceptance, and a sense of belonging, are vital for social inclusion and well-being of Individuals with serious mental illness (SMI). The mixed methods review critically explored multifaceted approaches supporting people with SMI to foster meaningful (non-intimate) social relationships or connections.

**Methods:**

Searches of eight electronic databases returned 4882 records. Duplicate removal, title abstract, then full-text, screening and hand searching resulted in 23 records for inclusion. Studies were integrated using the CIVIC Framework emphasising the importance of Closeness, Identity, Valued relationships, Involvement, feeling Cared for and accepted.

**Results:**

The review identified emotional and physical challenges, societal stigma, and other environmental factors to hinder making meaningful connections. Studies highlighted the necessity for interventions being adaptable, personalised, and encompassing of structured activities, peer and professional supports, and technology-assisted platforms.

**Conclusion:**

The complexity of social interactions for those with SMI call for comprehensive, holistic strategies to nurture social relationships within their communities.

**Supplementary Information:**

The online version contains supplementary material available at 10.1007/s00127-025-02812-8.

## Introduction

Meaningful connections are crucial for the mental health and well-being of individuals with serious mental illness (SMI). Adopting the World Health Organization [1] definition of mental disorders, in this paper SMI is operationalised as long-term mental illnesses involving substantial functioning impairment over multiple symptom and life domains. A useful definition for meaningful connections is relationships that provide people with emotional support, understanding, acceptance, sense of hope, purpose and belonging [2–4], with family, friends, or community.

Existing review studies show that social interaction, connection and personal relationships are crucial for people with SMI, with meaningful connections as a key aspect of recovery [5, 6]. Several empirical studies, however, show that the social networks of individuals with SMI are limited. A study of five European countries showed individuals with SMI (n = 7302) to have an average of three social contacts a week [7]. Another study showed adults with schizophrenia or schizoaffective disorder (n = 137), living in England, typically having one or two friends, usually other service users [8]. Loneliness and exclusion may increase the occurrence or severity of symptoms [9–11], whereas the quantity of social contact has associations with improved clinical and cognitive functioning, quality of life, and self-esteem among people with SMI [12, 13]. Okruszek, Piejka [14] showed that loneliness and symptom severity lead to increases perception of social threat. Threat compromises capacity to trust heightening fear of negative judgment [15–19]. McBride and Preyde [20] studied youth (n = 239) finding nearly half experienced bullying and preferred seclusion, with many worrying about problems going back to school following psychiatric hospitalisation. Research showed significant correlation between social behaviours of people with SMI and premature death [21], highlighting concerns with social seclusion and the importance social connections.

Community programs, therapeutic support groups, recreational and arts programs, or educational workshops, offer people with SMI opportunities to expand social networks, reducing loneliness [22]. Research shows community programs as particularly effective through offering socially safe spaces to connect with others during recovery processes [23]. A USA study [24] found that active involvement in community reduced depression and loneliness in populations with SMI. An integrative review spanning nine countries noted community belonging, trust, and hope as crucial for reducing loneliness and aiding recovery [25]. Zheng, Zhang [22] showed that meaningful community connections enriched life experiences, contributing considerably to mental health recovery.

Befriending programs, as one example, typically pair volunteers with individuals with SMI to undertake social activities. Review evidence from Australia, UK, and USA show both increases in friendships and goal-oriented outcomes [26]. Two befriending reviews showed modest effect on depressive symptoms and emotional distress, although effect size varied across populations [27, 28]. However, Farcas, Campbell [29] showed differences in social and clinical outcomes in schizophrenia, associated with befriending, due to diverse patient needs. Brooks, Devereux-Fitzgerald [30] stated that isolating interventions responsible for intervention effectiveness was challenging, calling for new ways to explore how to support meaningful socialisation among people with SMI. Accordingly, the current review broadens scope to explore individual and group interventions supporting meaningful connections.

This review is framed around the five CIVIC Framework dimensions, proposed by Hare Duke, Dening [31]: closeness, identity and common bond, Valued relationships, Involvement, and Cared for and accepted. ‘Closeness’ involves emotional proximity and mutual dependence. ‘Identity and common bond’ focus on shared identities within groups and fostering a sense of belonging. ‘Valued relationships’ emphasises the positive appraisal of interpersonal connections. ‘Involvement’ pertains to the degree of social engagement and activity participation. ‘Cared for and accepted’ covers aspects of social support and recognition, as integral to feeling part of a community. Each dimension contributes to what may constitute effective support to people with SMI in fostering meaningful connections [31, 32].

The CIVIC framework was chosen for its structured approach to understanding the complexity of social connections of people with SMI, categorizing social relationships into five distinct, interrelated dimensions. Categories assist identification of interventions with potential to enhance quality and depth of social connections; thereby, guiding intervention development when the constituents of meaningful interpersonal relationships become known. This leads to the review question: ‘In applying the CIVIC framework [31], what can be understood as the essential components of community-based interventions supporting people with SMI to make meaningful connections with others?’.

## Methods

This narrative systematic review was registered with PROSPERO (CRD42023454282) and conducted in accordance with the preferred reporting items for systematic reviews and meta-analyses (PRISMA) 2020 statement [33] (see S1 Supplementary File, Prisma Checklist).

### Eligibility criteria

The population, intervention, comparison, outcome, study (PICOS) tool operationalised the research question. The systematic review focused on people with SMI, encompassing youth (commonly > 15 years of age) and adult samples. Both were included to enable comparison, intervention tailoring, and holistic perspectives based on lifespan development, pending identification of relevant studies for inclusion.

A breadth of conditions (schizophrenia, bipolar disorder, PTSD, major depression, personality disorders, OCD, and eating disorders), and a breadth of interventions designed to promote meaningful social connections among people with SMI, were included. To undertake narrative synthesise of outcome effectiveness of ‘making connection’ interventions in the community, quantitative, qualitative, and mixed methods studies reporting original, primary research in English language academic journal articles were included. Results were extracted and considered in accordance with the dimensions of social connectedness in the CIVIC Framework [31]. Studies not focused on social connectedness, whole or in part, non-primary research, unpublished studies, and non-English texts were excluded. No publication date limiters were applied. Studies of intimate relationships were outside the scope of this review.

### Search strategy and screening

The search strategy was devised collaboratively with a specialist librarian. Eight electronic journal databases, CINAHL, the Cochrane Library, MEDLINE, ProQuest, PsycINFO, PubMed, Scopus, and Web of Science, and the first 10 pages of Google Scholar were systematically searched during December 2023. Backward and forward citation searches of records marked for inclusion were searched in January 2024. Keywords included (“social connectedness” OR companionship OR relatedness OR “sense of belonging” OR “social belonging” OR “group membership” OR “group identi*” OR “social identi*”) AND (“severe mental illness” or “serious mental illness” or smi or schizophrenia or psychotic or psychosis or ptsd or post-traumatic or mdd or “major depressi*” or bipolar or schizoaffective or “eating disorders” or schizoid or “personality disorder” or ocd or “obsessive compulsive”) AND (intervention or program or assistance or support or service). This was modified according to requirements of each database (Table S2 Supplementary File).

**Fig. 1:**
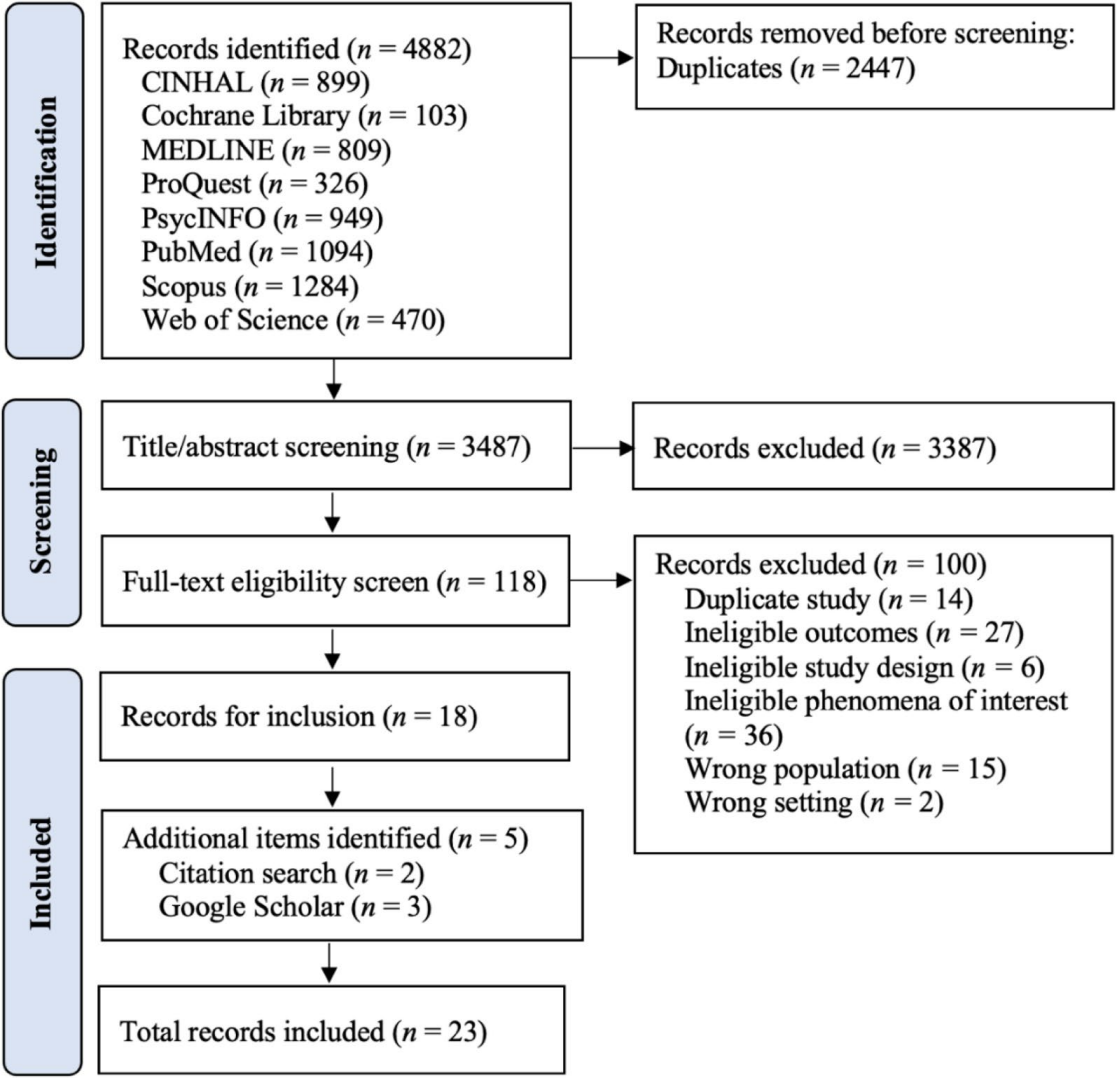
PRISMA flow diagram of studies included

Searching identified 4882 records. Upon exporting to Covidence (https://app.covidence.org), 2447 duplicates were automatically removed. Two-author independent screening (EP,HM) of titles and abstracts facilitated 3387 exclusions. Full-text double screening (EP,HM) of 118 records resulted in 18 records for inclusion, plus five records through forward/backward searching. All records were from discrete studies, representing 23 included studies (Fig. 1).

### Quality assessment

The Mixed Methods Appraisal Tool (MMAT) for quality assessment was chosen for its capability to evaluate various study designs; qualitative, quantitative, and mixed methods [34, 35]. One researcher (EM) quality assessed participant characteristics, objectives, methodologies, findings, and significance, employing the MMAT ‘yes, no, can’t tell’ scale (Table 1), checked by two authors (HM,YH).

**Table 1:** Mixed methods appraisal tool (MMAT)

Qualitative studies	Is the qualitative approach appropriate to answer the research question?	Are the qualitative data collection methods adequate to address the research question?	Are the findings adequately derived from the data?	Is the interpretation of results sufficiently substantiated by data?	Is there coherence between qualitative data sources, collection, analysis and interpretation?	Score
Bennett and Starnino [36]	Y	Y	Y	Y	Y	5/5
Boydell et al. [37]	Y	Y	Y	Y	Y	5/5
Burn et al. [38]	Y	Y	Y	Y	Y	5/5
Rice et al. [39]	Y	Y	Y	Y	Y	5/5
Carless and Douglas [40]	Y	Y	Y	Y	Y	5/5
Cassidy et al. [41]	Y	Y	Y	Y	Y	5/5
Dobbins et al. [42]	Y	Y	Y	Y	Y	5/5
Hogg et al. [43]	Y	Y	Y	Y	Y	5/5
McCorkle et al. [44]	Y	Y	Y	Y	Y	5/5
Naslund et al. [45]	Y	Y	Y	Y	Y	5/5
Pernice et al. [46]	Y	Y	Y	Y	Y	5/5
Prince et al. [47]	Y	Y	Y	Y	Y	5/5
Saavedra et al. [48]	Y	Y	Y	Y	Y	5/5
Valentine et al. [49]	Y	Y	Y	Y	Y	5/5
Yilmaz et al. [50]	Y	Y	Y	Y	Y	5/5
Wasylenki et al. [51]	Y	Y	Y	Y	Y	5/5
Quantitative studies	Are the participants representative of the target population?	Are measurements appropriate regarding both the outcome and intervention (or exposure)?	Are there complete outcome data?	Are the confounders accounted for in the design and analysis?	During the study period, is the intervention administered (or exposure occurred) as intended?	Score
Breitborde et al. [52]	Y	Y	CT	N	Y	3/5
McCorkle et al. [53]	Y	Y	N	CT	Y	3/5
Mixed methods studies	Is there an adequate rationale for using a mixed methods design to address the research question?	Are the different components of the study effectively integrated to answer the research question?	Are the outputs of the integration of qualitative and quantitative components adequately interpreted?	Are divergences and inconsistencies between quantitative and qualitative results adequately addressed?	Do the different components of the study adhere to the quality criteria of each tradition of the methods involved?	Score
Boyd [54]	CT	Y	Y	CT	CT	2/5
Harley et al. [8]	Y	CT	CT	CT	Y	2/5
Myers et al. [55]	Y	Y	Y	CT	Y	4/5
Wong et al. [56]	CT	Y	Y	CT	CT	2/5
Snethen et al. [57]	Y	Y	CT	CT	Y	3/5

### Narrative synthesis

Narrative synthesis followed the Economic and Social Research Council guidelines [58], beginning with preliminary tabulation of data using the MMAT checklist. Most studies were qualitative; therefore, results were synthesised via a thematic analysis of each study. Thematic analysis following Braun and Clarke [59] involved iterative reading for familiarisation, then code generation (EP,HM). Codes were grouped into themes, reviewed for accuracy, defined and named (EP,HM). A concept map was generated, upon which we identified the CIVIC Framework [31] as closely aligned to our inductive themes, offering a structure for reporting.

### Findings

This review included 23 studies, published from 1998 to 2022: 16 qualitative, 2 quantitative, and 5 mixed methods. Ten studies were from the USA, 6 UK, 3 Canada, 2 Australia, 1 Sweden, and 1 global sample via YouTube. The studies collectively had 964 participants, ranging from 5 to 154 participants, with ages ranging from 15 to 70 years. One study was youth-specific, targeting ages 15 to 35 with first episode psychosis, while the rest focused on adults aged 18 and above. Fifteen studies featured interventions aimed at improving social connections or addressing specific needs in people with SMI, while 8 were observational focusing on understanding social dynamics with no interventions (Table 2). Studies broadly conceptualised ‘social connections’ as any type of interpersonal relationship, covering friendships, family ties, community involvement, and connections formed through therapeutic or creative activities. Approximately 78% (18 of 23) of studies utilized broader interpretations of social connectiveness as any form of interpersonal relationship supporting individuals’ social and emotional well-being. The remaining 22% (5 of 23) examined quality, impact, and challenges specific to the friendships of people with SMI [8, 37, 47, 50, 56]. These studies offered broad perspectives on the experiences and interventions relevant to people with SMI across cultural and social contexts.

**Table 2:** Overview of included studies

	Authors, year Country	Aims	Participants	Methods	Findings
1	Bennett and Starnino [36]—United States	To explore personal experiences and perceptions of individuals with complex interpersonal trauma who participate in yoga	Five women with complex interpersonal trauma, aged mid-twenties to early sixties. Each participant had been engaging in a yoga practice at least once per week for the past three months. Participants were selected through purposive sampling after recruitment flyers were shared by five yoga teachers in an urban area of a Midwestern state	Design: qualitative phenomenological approach Intervention: a variety of yoga styles, with restorative practice and a more energetic practice Data collection: in-depth narrative interviews via Zoom Data analysis: guided by transcendental phenomenology starting from horizontalisation stage to a textural-structural synthesis	Four major themes were identified: (1) Transformation through yoga: it includes changes in emotional, mental, and spiritual states before, during, and after yoga sessions. (2) Comparing holistic benefits through opposing yoga styles: participants engaged in both restorative practices and more energetic practices, highlighting how different yoga styles cater to different aspects of healing and personal growth. (3) Community and relationships: it emphasises the connections formed with yoga teachers and fellow practitioners, and the supportive environment of the yoga community. (4) Trauma healing: it includes the personal accounts of how yoga has helped participants process and heal from their traumatic experiences
2	Boyd [54]—Australia	To create and assess a joint filmmaking initiative, engaging youths with severe mental health conditions in Victoria’s Grampians region, Australia	Six participants with SMI in Ballart, Victoria (4 males, 2 females) aged 18 – 26. The project collaborated with the KickStart Program to identify potential participants. An informational meeting held by the service attracted nine individuals, out of whom seven initiated the program, one person withdrawing after the first session	Design: convergent mixed-methods research Intervention: video-making workshops (Twice weekly for 5 weeks), outdoor filming at various locations, two short videos Data collection: participants were assessed using Sense of coherence scale, social Connectedness scale, subject quality of life scale. Post-program interviews were conducted by the author Data analysis: quantitative – comparing individual change scores against normative data and mean scores pre- and post-program using SPSS Version 13. Qualitative—thematic analysis	Quantitative: results showed statistically significant improvements in social connectedness and subjective quality of life post-program, as indicated by paired sample t-tests Qualitative: results, derived from thematic analysis of interviews, revealed themes of increased social interaction, improved motivation and well-being, enhanced creativity, increased confidence, and skill development among the participants. The program provided insights into mental health, changed perceptions on public attitudes, and although it faced some challenges for those with psychotic symptoms, it was generally well-received by the participants
3	Boydell et al. [37]—Canada	To explore the subjective meanings of friendship as described by people with SMI	Twenty-one individuals ranging in age from 27 to 61 years, were predominantly female (67%). Participants were enlisted through advertisements placed at outpatient clinics of a mental health centre. They had been diagnosed with SMI for durations ranging from 2 to 32 years	Design: qualitative, exploratory design Intervention: none Data Collection: semi-structured interviews were conducted, allowing participants to share their personal experiences and perspectives on friendship Data analysis: a narrative thematic analysis was utilised	Friendship is of profound importance to individuals with psychiatric disabilities, often impacting their mental health The participants faced unique challenges in forming and maintaining friendships, influenced by factors like stigma and the fluctuating nature of mental illness Despite these challenges, friendships provided crucial emotional support, helping to mitigate feelings of isolation and loneliness
4	Breitborde et al. [52] United States	To explore whether a specialised care program for those with first-episode psychosis significantly improves their satisfaction of essential psychological needs such as autonomy, competence, and social belonging	37 participants with first-episode psychosis, predominantly male. Participants were sourced from the Early Psychosis Intervention Center (EPICENTER). The participants met specific criteria: a diagnosis of schizophrenia-spectrum or affective disorder with psychotic features, first onset of psychotic symptoms within the past five years, ages 15 to 35, and a premorbid intelligence quotient over 70	Design: a non-randomised design Intervention: personalised therapy, medication management, and family support Data collection: were collected using the Basic Psychological Needs scale, before and after the program. Data analysis: repeated measures ANOVA to compare pre- and post-intervention scores	The study’s findings indicate significant improvements in the basic psychological needs of autonomy, competence, and relatedness among participants after six months of specialised care. Specifically: Autonomy: increased from a mean score of 4.14 at enrolment to 4.66 at the 6-month follow-up Competence: improved from a mean score of 3.82 at enrolment to 4.34 at the 6-month follow-up Relatedness: rose from a mean score of 4.21 at enrolment to 4.82 at the 6-month follow-up
5	Burn et al. [38] – United Kingdom	To understand the experiences of patients and volunteer befrienders participating in a befriending program designed for adults with psychosis	62 participants, comprising 34 volunteer befrienders and 28 patients. The study involved inviting individuals from a specific befriending program designed for adults with psychosis. The participants included both male and female adults, 18—65 of age	Design: qualitative descriptive design Intervention: befriending program for adults with psychosis Data collection: conducted through semi-structured interviews with both patients and befrienders Data analysis: thematic analysis was employed, enabling the identification of key themes that emerged from the interview narratives	Four key themes from the interviews: (1) Bridging the gap: the befriending program helped in reducing the social isolation experienced by patients, fostering connections between them and the wider community (2) A genuine relationship: the development of authentic, meaningful relationships between befrienders and patients over time (3) A big commitment: the commitment and challenges faced by the befrienders, highlighting the dedication required to make a positive impact (4) A flexible approach: The importance of flexibility in the program, accommodating the varying needs and circumstances of the participants
6	Carless and Douglas [40] United Kingdom	To understand how men with SMI experience social support within the context of their participation in exercise and sport	11 men from 24 to 43 of age, who had been diagnosed with SMI. Recruitment was based on the willingness to participate, assessments by mental health professionals, and their experiences in a variety of exercise or sport activities	Design: Qualitative ethnographic study Intervention: intervention that involved engaging men with SMI in various exercise and sport activities Data collection: participant observation, interviews and focus groups Data analysis: categorical content analysis using quotations as the unit of analysis	Four main themes: (1) Informational support: participants were provided with practical information about the benefits and availability of sport and exercise programs (2) Tangible support: this support was characterised by providing practical aids such as financial assistance and transportation. It addressed the physical barriers to participation (3) Esteem support: esteem support involved bolstering participants’ confidence and self-worth, which was particularly valuable during the early stages of involvement in sport and exercise 4) Emotional Support: it came from family, friends, and the sport community, providing comfort and a sense of belonging
7	Cassidy et al. [41] United Kingdom	To delve into the motivations and experiences of volunteers and befriendees participating in various UK-based befriending programs	38 befriending volunteers and 23 individuals, age 18 + , with SMI receiving befriending services encompassing eight voluntary sector organisations and four NHS entities. The selection was facilitated through referrals from the volunteer coordinators of each organisation	Design: qualitative descriptive study Intervention: one-on-one befriending program Data collection: participants answered questions from a semi-structured interview schedule and researchers followed up on interesting points and probed for further information where appropriate Data analysis: thematic analysis using inductive approach. The analysis was facilitated by using NVivo 11 software	The study identified three main themes: (1) Personal growth and altruism as motivations for volunteering: diverse motivations ranging from personal benefits, such as career advancement and gaining new experiences, to more altruistic reasons such as contributing to society and making amends for past actions (2) Benefits for befriendees through passive and active means: various benefits included enjoying simple conversations and gaining new perspectives from their befriender (3) Negotiating between professional role and friendship: the complexities of the befriender role required balancing the expectations of the volunteering organisation, the befriender’s own beliefs, and the needs of the befriendee
8	Dobbins et al. [42] United States	To explore the impact of group exergame play on the psychosocial health of older adults with SMI	16 older adults with SMI. Participants were recruited by the transitional residential treatment facility staff and referred to the researchers. Participants had an average age of 58.8 years, with a gender distribution of 62.5% male and 37.5% female. The racial composition included 50% White, 18.8% Black/African American, 6.3% Latinx, 12.5% Asian, and 12.5% other	Design: qualitative using grounded theory and symbolic interactionism Intervention: a 10-week program, playing active video games for 50 min thrice a week using Kinect for Xbox 360 Data collection: Focus groups and individual interviews. Observations, field notes, and coding memos were recorded, and researchers also participated in weekly group exergame activities Data analysis: using a constant comparison approach, involving transcription verification, and managed with Dedoose software	The key findings from this research include: (1) Social Connectedness: participants experienced improved social connectedness. The group setting and the nature of the exergames facilitated interactions and fostered a sense of belonging and support among the participants (2) Competence: the exergame intervention helped participants develop a sense of competence. Engaging in the games and overcoming challenges improved their self-efficacy and provided a tangible sense of skill acquisition (3) Positive social dynamics: playful competition enhanced group cohesion, belonging, and the ability to engage deeply in the games (4) Psychological well-being: the intervention positively impacted the participants’ psychological well-being, providing them with motivation, energy, and an opportunity to engage in physical activity in a socially supportive environment
9	Harley et al. [44] United Kingdom	To explore the nature of friendships in people with schizophrenia, focusing on both quantitative and qualitative aspects	137 participants with established schizophrenia or schizoaffective disorder, aged 18–65 Participants were recruited through community mental health teams in Southeast England	Design: a mixed-method survey design Intervention: none Data collection involved face-to-face interviews using semi-structured questions and standardised scales PANSS and SBS Data analysis: quantitative data were statistically analysed using SPSS software, while qualitative insights were drawn from personal accounts of friendships	Quantitative: the average number of friends reported by participants was 1.57. This number highlights the limited social networks among individuals with schizophrenia qualitative: the study identified three themes: (1) Emotional commitment: importance of emotional investment in maintaining friendships (2) Mistrust and stigma: challenges in forming friendships due to mistrust and perceived stigma (3) Friendship quality: despite having fewer friends, the quality of these friendships was generally reported to be good
10	Hogg et al. [43] United Kingdom	To explore how individuals experiencing psychosis perceive and navigate their social identities and group memberships	26 individuals experiencing psychosis range 18–45 years of age. Diverse group in terms of age, gender, and experiences	Design: qualitative descriptive design Intervention: none Data collection: semi-structured interviews to gather personal narratives Data analysis: thematic analysis to systematically identify and interpret patterns and themes within the interview data	Several key themes related to how individuals with psychosis experience and navigate their social identities: (1) Impact of psychosis on social identity: explores how experiencing psychosis affects individuals’ perceptions of themselves and their social groups (2) Social connections and well-being: highlights the significance of social relationships and group memberships in influencing the well-being of individuals with psychosis (3) Identity compartmentalisation: discusses how individuals manage and compartmentalise their social identity in the context of living with psychosis
11	McCorkle et al. [53] United States	To investigate the effectiveness of the Compeer program in enhancing social support for people with SMI	154 adults, with 79 assigned to the treatment group and 75 to a wait-list comparison group. 87% completed the 12-month follow-up assessment​​. Participants were recruited from 2001 to 2004 based on criteria: 18 + years old, having SMI, expressing interest in receiving Compeer services. Female (81%) and Caucasian (84%) individuals, with average age 45, 54% were single, 9% married, and 32% divorced or separated	Design: a non-randomised controlled study Intervention: compeer model of intentional friendship Data collection: baseline data was collected through individual interviews with participants within one month of being matched with a volunteer. These interviews were repeated after 6 and 12 months Data analysis: two-way analysis of variance (ANOVA) to compare the experimental and control groups based on their 6 and 12-month follow-ups	There was a significant increase in both social support and subjective well-being for those in the treatment group who responded to the Compeer intervention Those classified as “treatment responders” in the treatment group exhibited a significant reduction in psychiatric symptoms, especially depressive symptoms, from baseline to 12 months
12	McCorkle et al. [44] United States	To explore the experiences and perceptions of participants in the Compeer program for individuals with SMI	20 participants, which included nine adult clients and 12 volunteers. The recruitment strategy focused on individuals involved with the Compeer program. Nearly all participants being 30 + yo and some being of retirement age	Design: a qualitative design using grounded theory Intervention: compeer program, a volunteer friendship program for people with SMI Data collection: gathered through individual hour-long interviews conducted by two interviewers, each recording 10 interviews Data analysis: used preliminary descriptive methods, applying analytic techniques informed by grounded theory	The three themes: 1) Subjective descriptions of the relationship: they viewed their relationships with volunteers as social relationships that encouraged them to be more active outside their homes, fostered sociability and politeness in social settings, and stimulated intellectual engagement 2)Benefits of participating: it included improvements in mental health, social support, and overall well-being 3)Drawbacks of participating: this involved the emotional or time commitments required by the program​​
13	Myers et al. [55] United States	To analyse and articulate the concept of a ‘meaningful day,’ considered as one of the central elements in the recovery process for individuals with SMI	100 participants at baseline (18–65 years of age), with follow-up assessments conducted at 4, 8, and 12 months. The qualitative part of the study involved interviewing 30 individuals Participants were recruited from three Community Service Boards in the 34-county southeast region of Georgia	Design: a convergent mixed-methods design Intervention: opening Doors to Recovery program Data collection: participants’ self-assessments using a ‘Meaningful Day Thermometer’ and the number of meaningful days in the past month, along with ratings by professionals. Interviews were conducted using a semi-structured guide and focused on the meaningful day construct Data analysis: linear mixed models were fitted with time as a factor and generating codes from the transcribed interviews to identify themes	Companionship: Participants, family members, and staff believed that experiencing companionship is a crucial aspect of a meaningful day. This includes spending time with others, fostering relationships, and engaging in social activities Productivity: the perception that being productive, accomplishing tasks, and working towards goals contribute to the sense of having a meaningful day Achieving Stability: a meaningful day is seen as contributing to an individual’s stability in their recovery process. This involves setting and achieving goals, having a routine, and ensuring basic needs are met Autonomy: the concept of a meaningful day was linked to promoting independence and personal responsibility in the recovery process Insufficient Resources: a lack of resources was identified as a major barrier to achieving it
14	Naslund et al. [45] No specific country	To observe how individuals with SMI interact with peers on YouTube, considering the risks of disclosure and the potential benefits of managing their recovery and providing support to others​​	19 videos and their associated 3,044 comments. The recruitment involved evaluating 1,100 potential YouTube videos, following recommended video lists on the platform. The average views for these videos were 19,786, and they were on YouTube for 87 to 1,798 days. The video creators were mostly young adults (ages 18–35) and some middle-aged adults (ages 36–55)	Design: an ethnographic approach, using YouTube as a field Intervention: none Data collection: searched YouTube for publicly available videos. The first 100 videos for search terms: “mental illness,” “schizophrenia,” “schizoaffective disorder,” and “bipolar disorder” were screened Data analysis: the study used ATLAS.ti software for thematic analysis of comments	The study found that YouTube serves as a platform for peer-to-peer interactions and sharing personal experiences related to living with SMI. It highlighted how social media is reshaping how individuals with SMI engage with their environment and seek and share mental health care advice. The findings suggest potential benefits like learning from others, feeling supported, and forming relationships
15	Pernice et al. (46) United States	To explore the motivations of individuals with (SMI) for attending community programs known as “clubhouses.”	143 members from 10 clubhouse programs. The recruitment strategy was centred around these clubhouses, which offered a structured environment for members to engage in various activities, providing a sense of belonging and purpose	Design: a qualitative study Intervention: the clubhouse model—a community-based recovery approach offering structured activities and social support Data collection: semi-structured interviews Data analysis: using SPSS for Mac for thematic analysis	Several benefits associated with clubhouse membership, including lower hospitalization rates and healthcare costs, higher quality of life scores, and greater success in supported employment compared to matched control groups. Clubhouse members reported experiencing a robust sense of community, which translated into higher self-empowerment ratings
16	Prince et al. [47] United States	To investigate how individuals with SMI form close relationships, focusing on the challenges and facilitators of such connections	20 participants with SMI. The recruitment strategy involved enlisting participants from a community mental health agency, known as Fountain House which simulated a daily work-day activity and schedule	Design: qualitative descriptive study Intervention: none Data collection: focus groups and audio recorded Data analysis was thematic, focusing on identifying patterns and key themes related to the complexities of forming and maintaining close relationships	The study identified four main themes: (1) The complex impact of severe mental illness on the capacity to form close relationships, highlighting how symptoms can both hinder and motivate social connection; (2) The crucial role of shared experiences, particularly within mental health programs, in fostering a sense of belonging and understanding; (3) The significant barriers to forming relationships, including societal stigma and personal insecurities; and (4) Strategies for overcoming these barriers, such as leveraging support groups and focusing on common interests to build connections
17	Rice et al. [39] Canada	To evaluate the effects of long-term group for people with schizophrenia on qualitative changes in their interactions and overall mental health	32 participants in a long-term outpatient group therapy for durations spanning up to 28 years. Participants were recruited through a psychiatry outpatient clinic	Design: a qualitative descriptive design Intervention: not specific, regular group therapy sessions at an outpatient clinic Data collection: observing and documenting qualitative changes in group interactions, personal experiences, and hospitalisation rates over a period of up to 33 years. Data analysis: by reviewing and identifying significant trends and patterns in the participants’ experiences and behaviours throughout the program	A significant increase in supportive comments among group members, indicating improved social interactions and empathy A noticeable decrease in hospital admissions among the participants, suggesting an improvement in managing their condition Significant personal growth and coping strategies among members, reflecting the therapy’s positive impact on their lives The ability of group members to cope with crises, such as personal losses, without necessitating hospital admission, was observed
18	Saavedra et al. [48] United Kingdom	To evaluate the impact of participating in creative workshops on social connectivity, psychological well-being, and subjective experience of people SMI	19 individuals including both service users and key workers. They were chosen through their involvement in creative workshops held at a museum	Design: qualitative descriptive design Intervention: creative workshops held in a museum setting, aiming to engage participants in artistic activities Data collection: a combination of interviews, participant observations, and group discussions Data analysis: using Atlas-Ti software to identify and interpret significant themes	The five key themes (1) Learning process: participants experienced enhanced cultural knowledge and developed critical thinking skills (2) Social connection: there was a notable improvement in social interactions and the expansion of social networks among the participants (3) Psychological well-being: the workshops contributed to increased overall well-being, stress relief, and positive changes in mood (4) Institutional change: the study observed shifts in professional practices and approaches to healthcare delivery (5) Mutual recovery: both service users and healthcare professionals experienced reciprocal benefits, emphasising a shared journey of recovery
19	Snethen et al. [57] United States	To evaluate the effectiveness of the I-CAN intervention in increasing community involvement, planning, and coping skills among adults with schizophrenia	10 individuals with Schizophrenia Spectrum Disorders initially enrolled, 8 participated. Participants were recruited through a targeted strategy aimed at adults meeting specific diagnostic criteria, without communicable diseases that hinder community engagement	Design: a pilot study – mixed methods, Intervention: a novel recreational-therapy program designed to boost community engagement and coping strategies among adults with schizophrenia. Data gathering involved a combination of interviews, therapist observations, and participant diaries over a 10-week period. Analysis was carried out using a thematic approach to identify key outcomes and insights from both qualitative and quantitative data streams	The study identified significant enhancements in community participation and coping abilities among participants. It distilled these outcomes into several themes: (1) Increased engagement in community activities, highlighting how individuals became more active and involved in their communities; (2) Enhanced planning and goal-setting skills, noting improvements in participants’ ability to plan and execute activities; and (3) Development of coping mechanisms, which emphasised how participants learned to better manage stress and challenges
20	Valentine et al. [49] Australia	To explore young people’s engagement with “Horyzons”, a social media-based intervention for first-episode psychosis	12 young individuals aged between 19 and 28 who participated in Horyzons. The recruitment strategy utilised a quantitative categorization based on platform usage levels of the 85 Horyzons RCT treatment group participants, leading to interviews with a diverse group representing varying degrees of engagement	Design: a qualitative design Intervention: Horyzons, a long-term, online social media-based intervention which combined therapeutic content, peer and professional support within a moderated online platform Data were collected through semi-structured interviews. Data analysis followed a thematic approach, using NVivo	The study identified three main themes: (1) The intervention as a unique support system that bridges the gap between professional help and everyday social interaction; (2) The platform’s role in enhancing participants’ understanding and management of psychosis; (3) Challenges and limitations related to engagement and platform dynamics. Each theme captures aspects of the intervention’s impact, from providing continuous support to navigating the complexities of engagement and the digital environment’s limitations
21	Wasylenki et al. [51] Canada	To explore the effectiveness of Social Network Therapy for individuals with schizophrenia	Four distinct cases within the Social Network Therapy Program. Case One involved a 23-year-old man having poor motivation and negative feelings toward family members. Case Two presented a 30-year-old female facing challenges with over-protective parents, stress at work, and a lack of close relationships. Case Three was a 30-year-old man, who had become dependent on his family while isolating himself from other supports. Case Four involved a 22-year-old female who expressed dissatisfaction with her social network, experiencing severe loneliness and shyness	Design: a case study design, Intervention: social network therapy for individuals with schizophrenia involving specialised training for therapists in network therapy practices Data collection: client interviews, structured interviews, rating scales, and therapists’ uniform “contact sheets” documenting interactions with clients Data analysis: qualitative assessments of the therapy’s impact on clients’ social networks and well-being, illustrated through case examples	Four main themes: environmental change: emphasises transitioning clients from restrictive, kin-dominated networks to broader, supportive social environments Relationship counselling: focuses on enhancing clients’ abilities to forge and sustain healthier, reciprocal personal relationships Therapeutic alliance: highlights the importance of building a cooperative working relationship between the therapist, the client, and their social network Readiness for change: considers the willingness and ability of clients and their networks to engage in and benefit from therapy
22	Wong et al. [56] United States	To examine the social integration of individuals with serious mental illness, focusing on their social network transactions and the satisfaction derived from these interactions	60 participants. These individuals were recruited through mental health service providers	Design: a mixed-methods study design Intervention: none Data collection involved structured interviews to gather data on social network transactions, and relationship satisfaction Data analysis included statistical methods such as ANOVA and t-tests, and thematic analysis for qualitative responses	Frequency of transactions: the average number of positive transactions per week was significantly higher than negative transactions Type of transactions: emotional support transactions were the most frequent, with a high average count per week. Tangible support and problem-solving transactions were less frequent but still notable Nature of interactions: positive interactions outnumbered negative ones across all types of transactions. The nature of these interactions varied significantly based on the relationship source (family, friends, service staff)
23	Yilmaz et al. [50] Sweden	To examine the ways in which individuals with schizophrenia engage socially during daily activities across various settings	12 participants, selected through a purposive sampling approach from a community mental health centre	Design: a qualitative study. Intervention: none Data were collected through field notes and video recordings Data analysis: thematic analysis process, where patterns, themes, and categories were identified and interpreted	Three main themes: (1) The role of environmental context, highlighting how physical and social settings influence interaction opportunities; (2) Interaction strategies, detailing the methods participants used to engage with others, including both proactive and reactive approaches; and (3) Barriers to social participation: symptoms of schizophrenia and societal stigma, which hindered their ability to interact socially

### Diverse interventions enhancing social connections

Studies encompassed a broad range of activities addressing the social needs of people with SMI. Interventions, such as yoga for trauma recovery [36], filmmaking projects [54], and specialised care programs [52], offered diverse pathways for engagement. From befriending programs [38, 41] fostering direct interpersonal connections, to innovative solutions such as social media-based interventions [49] and recreational therapy [57], these initiatives provided essential support structures. Physical activities, highlighted by Carless and Douglas [40] and Dobbins, Hubbard [42], emphasised the importance of exercise and groups in enhancing social connections and physical health. The Compeer intentional friendship making program [44, 53] and the Clubhouse model offering structured activities [46] focused on building supportive communities through peer relationships. Creative workshops [48] offered expressive outlets and social interaction, while the Opening Doors to Recovery program [55] and Social Network Therapy [51] applied structured frameworks for integrating people with SMI into the community. Interventions across studies were systematically organised, with peer and professional supports, tailored to individual interests and needs, fostering a sense of community belonging. In-person and digital platforms facilitated accessibility and engagement, demonstrating holistic approaches to mental health recovery and social integration.

Exploratory studies delved deeper into the aspects of social connections, focusing on social dynamics and interactions. Through examining the essence of friendships [8, 37], the role of digital platforms [45] and the supportive environments of clubhouses [47] illuminated multifaceted aspects of social integration. Healing impacts of long-term group therapy [39] and importance of everyday social interactions [50, 56] provided insights into the significance of community, collective experiences, and social challenges in fostering meaningful connections.

**Fig. 2:**
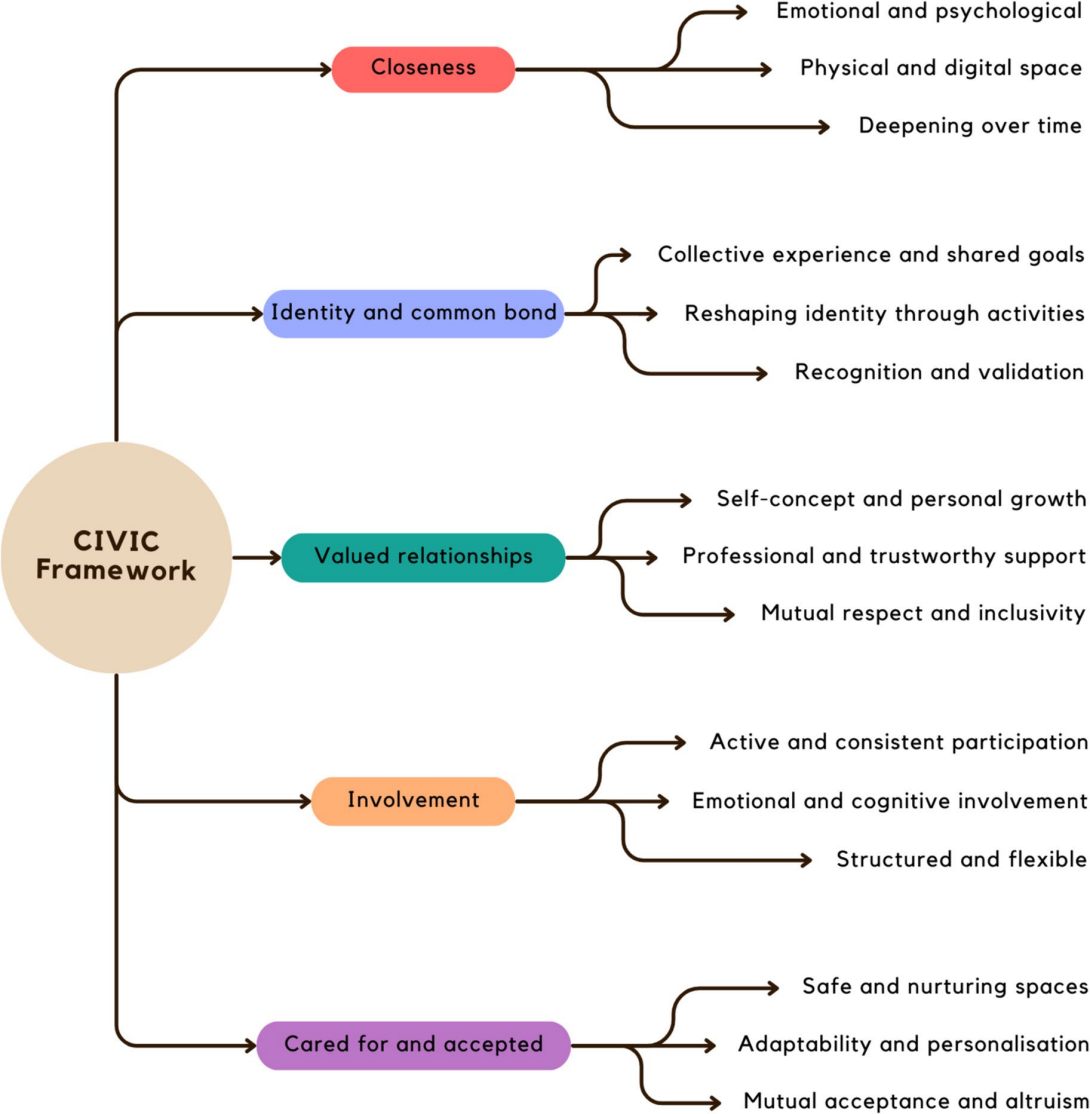
Applying the CIVIC framework to foster meaningful connections in SMI

Figure 2 presents a visual synthesis on social connections in people with SMI from review results, arranged according to the CIVIC Framework, illustrating ‘Closeness’ through themes of emotional bonds, shared spaces, and evolving relationships. ‘Identity and common bond’ are depicted by collective experiences, activity-based identity formation, and social recognition. The ‘Valued relationships’ dimension highlights personal growth, supportive professional relationships, and the essence of respect and inclusivity. ‘Involvement’ encompasses themes of active participation, emotional and cognitive engagement, and a balance between structure and adaptability in social activities. The final dimension, ‘Cared for and accepted,’ focuses on the creation of supportive environments, tailored personal interactions, and the fostering of altruism.

## Closeness

### Emotional and psychological closeness

People with SMI frequently face dual challenges of complex trauma and a profound sense of mistrust, manifesting in general social interactions and when engaging health professionals. Bennett and Starnino [36] studied five women who experienced complex trauma, finding that yoga provided a unique environment for the women to safely explore and reconcile their past traumas. Coupled with guidance from a trusted instructor, participation was instrumental in cultivating closeness and mentorship. Two studies shared strategies for developing emotional closeness: Rice, Zorn [39] with 32 participants attending a group therapy program in Canada for between 1–28 years, and; Burn, Chevalier [38] via a UK befriending program involving 34 volunteers and 28 patients. Both studies showed that safe and confidential spaces were crucial for building trust, while active and empathetic listening validated and supported peoples’ feelings, fostering environments characterised by emotional safety and closeness. Likewise, Cassidy, Thompson [41] with 38 volunteers and 23 individuals with SMI in the UK, emphasised that facilitating open and honest discussions about personal experiences, struggles, and successes allowed participants to share vulnerabilities. Closeness among participants manifested as a result.

### Physical and digital space closeness

In physical spaces, face-to-face interactions, nonverbal cues, eye contact and physical touch, played a role in fostering closeness, intimacy and trust. Harley, Boardman [8] highlighted that people with schizophrenia, with limited social networks, frequently maintained high levels of emotional commitment and support from their few friends. Boyd [54] and Prince, Ansbrow [47] explored how teamwork and collaborative activities contributed a sense of closeness among participants with SMI. Physical exercise, collaborative and creative projects, and social gatherings allowed people in several studies to form closeness (with each other) through direct engagement, shared experiences, and collective navigation of their physical environments [36, 40, 42, 48, 55, 57]. Naslund, Grande [45] extended this concept to online communities, highlighting digital platforms as vital spaces for emotional support and shared healing. Valentine, McEnery [49] explored technology-mediated activities (Horyzons, social media-based) and illustrated enhanced emotional resonance between young people navigating SMI. These findings, alongside observed social integration in clubhouse settings [46] and nurturing environments of creative workshops [48], underscored the value of intentional settings and activities that cultivated closeness, transcending traditional boundaries of therapeutic supports.

### Deepening closeness over time

The gradual evolution of friendships, in programs such as Befriending and Compeer, emphasised the dynamic nature of closeness in social support settings [38, 41, 44, 53]. Initially, these relationships were structured and formal, grounded in program guidelines and objectives. As befriending pairs meet regularly and share experiences, transformations were observed [38, 44]. Two studies showed that conversations flowed more freely, leading to less structured and spontaneous activities over time [41, 53]. Longer-term programs or interventions (> 1-year) with repeat interactions appeared responsible for relationships deepening, facilitating closeness [44]. Breitborde, Pine [52] documented people in a specialised support program to experience substantial increases in relatedness over the six months studied. Similarly, Myers, Smith [55] revealed that participants in the Opening Doors to Recovery program increasingly viewed their days with others as meaningful.

## Identity and common bond

### Collective experience and shared goals

Community-based group therapy ware instrumental in fostering a sense of belonging by uniting participants through shared experiences of navigating their struggles [44, 53, 54]. Sense of belonging was cultivated through creative projects, like filmmaking [54] or museum-based art workshops [48]. Common creative pursuits promoted teamwork and reinforced shared identity. Digital platforms expanded the reach of peer interaction via virtual space [43, 45, 49]. Cassidy, Thompson [41] showed that empathy and altruism of volunteers deepened connections between people with SMI, particularly when volunteers had personal or familial mental health experiences.

### Reshaping identity through activities

Involvement in activities significantly enhanced personal growth and identity formation. Boyd [54] and Saavedra, Arias [48] elucidated how shared interests in filmmaking and creative arts were a catalyst for developing positive identities. This transformative process was mirrored in sports and recreational programs, with two studies observing that collective engagement, teamwork, and mutual encouragement in physical activities engendered shared identity [40, 57]. Artistic activities in identity reconstruction was also noteworthy, with Saavedra, Arias [48] highlighting art workshops as powerful conduits for reshaping identities and relationships. The way that people navigate their mental health challenges significantly contributed to their social identity, as illustrated by several studies [37, 43, 47, 50]. They emphasised that supportive environments were pivotal in allowing people with SMI to reconstruct their identities in a manner that acknowledged their unique experiences and need for belonging.

### Recognition and validation

The concept of recognition and validation was central to developing shared identity and common bond among people in the studies reviewed. Yoga teachers were influential, underscoring the role of activity leaders in shaping identity through solidarity and shared experience [36]. Acknowledgment of collective struggle with mental health provided strong foundation for community belonging and personal growth [37]. Supportive feedback during sports and activity, recognising individual contribution, enhanced group bonding [40]. Artistic endeavours [48] and shared personal narratives [39], including through digital platforms [49], were other avenues where recognition and validation played a role in strengthening meaningful connections and communal ties.

## Valued relationships

### Self-concept and personal growth

Having valued relationships can improve self-concept and self-esteem. Yoga practitioners observed enhanced self-esteem and body positivity in people with SMI [36], while participants in creative activities reported increased confidence [48, 54]. This sub-theme captured the sense of achievement that comes from engaging in meaningful tasks in the presence of others during the recovery process [55], contributing to more profound interpersonal connections.

### Professional and trustworthy support

Professional support and trust were critical for developing valued relationships, particularly within therapeutic contexts. Trust in the guidance of physiotherapists [40], the transformative perception of psychosis through befriending [38], and acknowledgment of professional boundaries [41] each contributed to perceptions of security. The therapeutic alliance in group therapy settings [39] and direct care [57], demonstrated the necessity of trust and professionalism in nurturing valued relationships.

### Mutual respect and inclusive environments

Mutual respect and inclusivity were recognised as foundational for cultivating valued relationships. Appreciation of mutual support within SMI communities [37], acceptance and understanding offered in non-judgmental spaces [47], and social comfort derived from shared clubhouse activities [47], promoted inclusive atmospheres where relationships could thrive. Whether digital [49] or community-based [48], inclusivity allowed for exchange of support and advice between people that fostered connections respectful and accepting of individual needs and preferences [50, 56].

## Involvement

### Active and consistent participation

Several studies showed that routine, as with yoga classes, sports, creative group activities, and group programs, motivated active involvement [36, 39, 54, 57]. Compeer [44, 53] demonstrated that regularity of engagement was essential for strengthening relationships and fostering community ties. Horyzons [49] digital program and clubhouse activities [46, 47] showed associations between consistent involvement, sustained commitment, and social and personal development.

### Emotional and cognitive involvement

Emotional engagement was reflected in the deep connections formed through shared experiences and mutual (peer) support, in mental health systems [37] and group settings [42]. Cognitive involvement took place thorough active learning and skill acquisition workshops [48], and sports programs [40]. Emotional and cognitive outcomes cross-linked with the *Self-concept and personal growth* theme, since personal development was seen to strengthen confidence, self-esteem, and sense of self. Bennett and Starnino [36] elucidated the processing of thoughts and experiences in healing journeys, interlinking emotional and cognitive involvement with fostering socialisation and personal growth.

### Structured and flexible activities

Balance between structure and flexibility was found crucial for facilitating participant involvement. At Fountain House, participants and staff collaborated closely in routine and purpose, simulating activities and schedules of typical workdays. This provided comfortable spaces for engagement [47], while flexible activities offered personalised levels of involvement [50]. The varied involvement of volunteers in professional and friendship roles [41] showed adaptability in meeting individual needs within structured programs. Moreover, capacity to maintain involvement despite internal struggles or social stigma [49] highlighted the importance of flexibility in supporting active participation in varied social roles and activities [51].

## Cared for and accepted

### Safe and nurturing spaces

Participants across programs consistently highlighted how program conveners created safe spaces, fostering feelings of being cared for and accepted [36, 39, 46]. Such environments allow people to express themselves without fear of judgment, contributing to a sense of security and emotional support as crucial for those with mental health challenges [8, 43].

### Adaptability and personalisation in care

The adaptability and personalisation of care were evident in the approaches of activity instructors, therapists, and volunteers [36, 38, 52]. Flexibility to adapt and personalise care confirmed the value placed on each individual, contributing to their sense of being understood and supported [40, 41].

### Mutual acceptance and altruism

Mutual acceptance and altruism shaped the interactions within programs, relationships among participants, and relationships between participants and facilitators. This dynamic was observed in sharing personal stories [45, 54], collaborative art projects [48], and in the deep bonds formed in volunteer-client pairings [44, 53]. Activities emphasised the critical role of reciprocal understanding and support, via interactions between people with and without SMI demonstrating shared commitment toward each other’s well-being, indicative of valued and altruistic relationships [50, 56]. Wong, Matejkowski [56] detailed moderate satisfaction with social relations and regular network contact, indicating feeling cared for within one’s social circle was beneficial.

## Barriers in forming and sustaining meaningful connections

The formation of meaningful connections was impeded by a spectrum of barriers, as evidenced in several studies. Emotional regulation and physical limitations posed significant obstacles within therapeutic practices [36]. Boyd [54] emphasised the impact of managing psychotic symptoms requiring individualised support. Others showed that stigma subtly eroded social engagement of people with SMI [37]. The isolating effects of psychiatric symptoms limited social opportunities, further compounding this challenge [37, 38, 40]. Misaligned expectations between volunteers and people with SMI, along with logistical hurdles, often broke down social support systems [41, 42].

Fear of rejection and internalised stigma severely limited social interactions [8, 43]. Unsuccessful matching of volunteer friends, such as with Compeer, sometimes exacerbated social isolation and stigma [44, 53]. Online platforms, while beneficial, carried risk of misinformation which heightened distress, adding complexity to the social landscape for people with SMI [45]. Two studies reflected on the psychological barriers and the lack of structured environments that inhibit facilitation of connections [46, 47]. Institutional resistance and misconceptions about SMI were identified as leading to a dearth of authentic self-expression [39, 48]. Lastly, five studies showed that limitations in function and mobility, social anxiety, and poor social skills were significant impediments for people with SMI in building and nurturing valued relationships [49–51, 56, 57].

**Fig. 3:**
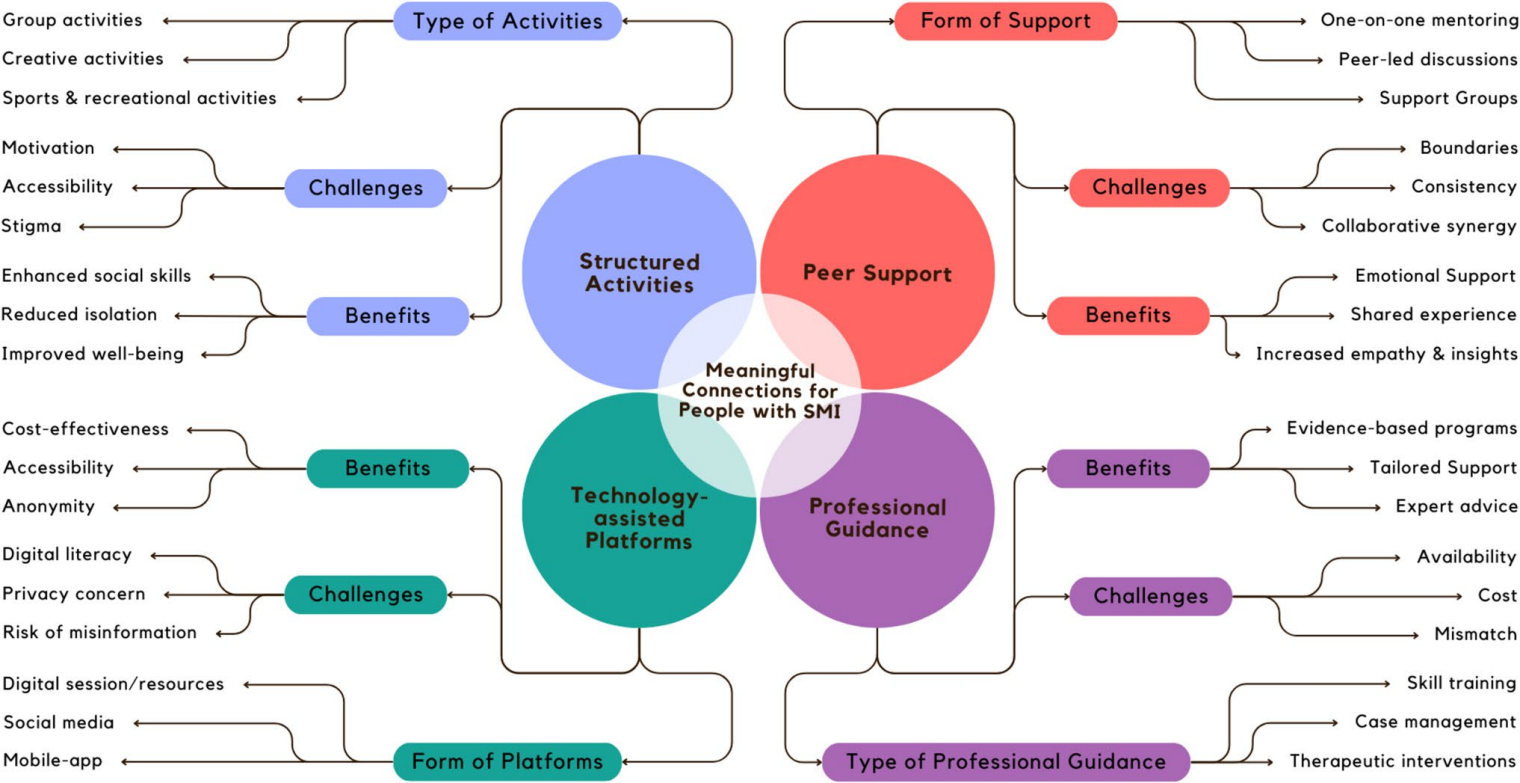
Conceptual framework illustrating core components of interventions for fostering meaningful connections for people with SMI

Drawing from the results of our review study, we crafted a conceptual map that delineates the fundamental elements necessary for interventions aimed at cultivating meaningful connections (see Fig. 3). This map, as a visual guide, articulates the interplay between structured activities, peer support, professional guidance, and technology-enhanced platforms. These components, garnered from the studies reviewed, seem pivotal in creating environments where people with SMI can socially thrive. They promote engagement, offer support, and facilitate access to community resources, enhancing social connections. They acknowledge peer support and shared experiences as instrumental for sense of belonging, while professional guidance ensures interventions are delivered effectively. Technology expands reach, offering new avenues for connection.

## Discussion

Findings from the 23 studies, integrated within the CIVIC Framework, offers evidence that the construction of meaningful connections and feeling about having a supportive community is multifaceted. The CIVIC Framework outlines a structured approach towards understanding and potentially addressing social connectedness in people with SMI through five dimensions: Closeness, Identity and common bond, Valued relationships, Involvement, and being Cared for and accepted [31, 32]. Our review offers empirical evidence or theoretical expansions that enrich the application of the CIVIC Framework to people with SMI in developing and maintaining meaningful connections that can extend to real-world settings.

This review established the concept of closeness as central to forming meaningful connections. It encompassed both physical and digital proximities, as well as the emotional and psychological connections that deepen over time. This closeness fosters environments where identity and common bonds can flourish, illustrated by collective experiences, reshaping identity through activities, and critical processes of recognition and validation. The concept of valued relationships emerged as a cornerstone, highlighting the interplay between self-concept, personal growth, and support provided by trustworthy and respectful professionals. Inclusive environments that fostered mutual respect (between participants, and between participants and program facilitators) encouraged the formation and sustaining of relationships. Involvement, another key theme, emphasised the importance of active, consistent participation, emotional and cognitive engagement, and structured yet flexible activities that catered to individuals. This involvement was not passive, but instead marked by active agency and meaningful engagement in community life. The sense of being cared for and accepted was paramount. Safe and nurturing spaces, adaptability and personalisation in care, and a culture of mutual acceptance and altruism were argued essential for people with SMI to make connections. This sense of mutual acceptance was fostered in specific community settings, such as clubhouses, support groups, and arts-based workshops [46, 48]. These structured yet nurturing environments provided safe spaces where individuals with SMI participated in shared activities, leading to feelings of being valued, respected, and understood. Such settings, noted by Boyd [54], could then translate to wider societal interactions, allowing people with SMI to extend the acceptance experienced in targeted community settings to their broader social encounters.

Forming meaningful connections with targeted interventions, such as befriending, compeer or clubhouse, provided structured pathways to engage socially, leveraging guided activities and peer support to foster relationships [38, 41, 44, 46, 53]. Interventions proactively tackled social isolation by providing spaces to engage with others who share similar challenges, thereby facilitating formation of supportive connections and networks. Conversely, forming connections without targeted interventions relied on natural social opportunities, such as chance encounters in community settings, public spaces, or online social media, which were less predictable and required greater individual effort [8, 37, 43, 45, 50]. While both approaches may facilitate meaningful relationships, targeted interventions were more deliberate and supportive, at least initially. Targeted interventions were suggested to make the process of connecting less daunting for people with SMI.

The visualisation of review evidence, shown in Fig. 3, underscores the importance of multi-faceted approaches for integrating direct interventions with supportive (and adaptable) environments to nurture meaningful connections, including in community spaces. Additionally, as a concept-framed review, results structured around the notion of social citizenship acknowledges benefits associated with having access to all of community’s resources to support recovery. While not a factor identified or emphasized in the studies reviewed, and not featured in Fig. 3, this does not negate the importance of having meaningful connections in the broader community for health and recovery.

As with all review studies, we acknowledge potential biases in study selection, interpretation, challenges assessing quality of included studies, and difficulty comparing studies due to heterogeneity in method and context. Youth and adult populations were included to compare experiences, interventions, and outcomes. Locating one discrete youth-focussed study made analytical insights not possible. These limitations may impact the review’s comprehensiveness and the generalisability.

## Conclusion

This review of interventions that nurture meaningful connections for people with SMI was structured using the CIVIC Framework. Review insights focused on types of activities, support, platforms, and professional guidance, including challenges, benefits, and synergies between them. Accordingly, the role of social connectedness in mental health was underscored, particularly interventions incorporating structured activities, peer support, professional guidance, and technology-assisted interventions. Future studies implementing rigorous methodologies, ensuring diversity and representativeness of study samples, are needed. As are standardised measures assessing longer-term effects of in-person and technology-assisted interventions on social connectedness, citizenship, and mental health outcomes for people with SMI.

## Supplementary Information

Below is the link to the electronic supplementary material.

Supplementary file1 (DOCX 32 KB)

Supplementary file2 (DOCX 21 KB)

## Data Availability

No datasets were generated or analysed during the current study.
