# Supplementary material for: Interventions supporting meaningful connections for people with serious mental illness: a concept-framed systematic narrative review
Source: Soc Psychiatry Psychiatr Epidemiol. 2025 Jan 18;60(6):1257–82. doi: 10.1007/s00127-025-02812-8 (PMC12162793; doi:10.1007/s00127-025-02812-8)
Supplement: Supplementary file 2 — Supplementary file2 (DOCX 21 KB) [file 127_2025_2812_MOESM2_ESM.docx]

**Supplementary 1**

**Search Strategy**

**CINAHL**

899 documents

| **#** | **Query** | **Results** |
| --- | --- | --- |
| 1 | "social connectedness" OR companionship OR relatedness OR "sense of belonging" OR "social belonging" OR "group membership" OR "group identi*" OR "social identi*" | 51,594 |
| 2 | "severe mental illness" or "serious mental illness" or smi or schizophrenia or psychotic or psychosis or ptsd or post-traumatic or mdd or "major depressi*" or bipolar or schizoaffective or "eating disorders" or schizoid or "personality disorder" or ocd or "obsessive compulsive" | 157,336 |
| 3 | intervention or program or assistance or support or service | 1,881,347 |
| 4 | 1 and 2 and 3 | 899 |

**Cochrane Library**

103 Trials matching "severe mental illness" or "serious mental illness" or smi or schizophrenia or schizophrenic or psychotic or psychosis or ptsd or post-traumatic or mdd or "major depression" or "major depressive" or bipolar or schizoaffective or "eating disorders" or schizoid or "personality disorder" or ocd or "obsessive compulsive" in Title Abstract Keyword AND "social connectedness" OR companionship OR relatedness OR "sense of belonging" OR "social belonging" OR "group membership" OR (group NEXT identi*) OR (social NEXT identi*) in Title Abstract Keyword AND intervention or program or assistance or support or service in Title Abstract Keyword - (Word variations have been searched)

**MEDLINE**

809 documents

| **#** | **Query** | **Results** |
| --- | --- | --- |
| 1 | "social connectedness" or companionship or relatedness or "sense of belonging" or "social belonging" or "group membership" or "group identi*" or "social identi*" | 46,235 |
| 2 | "severe mental illness" or "serious mental illness" or smi or schizophrenia or psychotic or psychosis or ptsd or post-traumatic or mdd or "major depressi*" or bipolar or schizoaffective or "eating disorders" or schizoid or "personality disorder" or ocd or "obsessive compulsive" | 491,367 |
| 3 | intervention or program or assistance or support or service | 12,227,144 |
| 4 | 1 and 2 and 3 | 809 |

**ProQuest**

326 documents

abstract("social connectedness" or companionship or relatedness or "sense of belonging" or "social belonging" or "group membership" or "group identi*" or "social identi*") AND abstract("severe mental illness" or "serious mental illness" or smi or schizophrenia or psychotic or psychosis or ptsd or post-traumatic or mdd or "major depressi*" or bipolar or schizoaffective or "eating disorders" or schizoid or "personality disorder" or ocd or "obsessive compulsive") AND abstract(intervention or program or assistance or support or service)

**PsycINFO**

949 documents

| **#** | **Query** | **Results** |
| --- | --- | --- |
| 1 | "social connectedness" or companionship or relatedness or "sense of belonging" or "social belonging" or "group membership" or "group identi*" or "social identi*" | 52,742 |
| 2 | "severe mental illness" or "serious mental illness" or smi or schizophrenia or psychotic or psychosis or ptsd or post-traumatic or mdd or "major depressi*" or bipolar or schizoaffective or "eating disorders" or schizoid or "personality disorder" or ocd or "obsessive compulsive" | 509,192 |
| 3 | intervention or program or assistance or support or service | 1,192 ,171 |
| 4 | 1 and 2 and 3 | 949 |

**PubMed**

1,094 documents

(("social connectedness" or companionship or relatedness or "sense of belonging" or "social belonging" or "group membership" or "group identi*" or "social identi*") AND ("severe mental illness" or "serious mental illness" or smi or schizophrenia or psychotic or psychosis or ptsd or post-traumatic or mdd or "major depressi*" or bipolar or schizoaffective or "eating disorders" or schizoid or "personality disorder" or ocd or "obsessive compulsive")) AND (intervention or program or assistance or support or service)

**Scopus**

1,284 documents

TITLE-ABS-KEY ( ( "social connectedness" OR companionship OR relatedness OR "sense of belonging" OR "social belonging" OR "group membership" OR "group identi*" OR "social identi*" ) AND ( "severe mental illness" OR "serious mental illness" OR smi OR schizophrenia OR psychotic OR psychosis OR ptsd OR post-traumatic OR mdd OR "major depressi*" OR bipolar OR schizoaffective OR "eating disorders" OR schizoid OR "personality disorder" OR ocd OR "obsessive compulsive" ) ) AND ( intervention OR program OR assistance OR support OR service ) AND ( LIMIT-TO ( LANGUAGE , "English" ) ) AND ( LIMIT-TO ( DOCTYPE , "ar" ) )

**Web of Science.**

470 documents

(("social connectedness" or companionship or relatedness or "sense of belonging" or "social belonging" or "group membership" or "group identi*" or "social identi*") AND ("severe mental illness" or "serious mental illness" or smi or schizophrenia or psychotic or psychosis or ptsd or post-traumatic or mdd or "major depressi*" or bipolar or schizoaffective or "eating disorders" or schizoid or "personality disorder" or ocd or "obsessive compulsive")) AND (intervention or program or assistance or support or service)
